# Introducing the Archive of Interwar Europe Election Data & Assemblies (AIEEDA)

**DOI:** 10.1038/s41597-025-04969-y

**Published:** 2025-04-15

**Authors:** Lea Kaftan, Bruno Della Sala, Olga Jerjomina, Stefan Stojkovic, Edoardo Alberto Viganò, Nils-Christian Bormann

**Affiliations:** 1 GESIS Leibniz Institute for the Social Sciences, 50667 Cologne, Germany; 2 Witten/Herdecke University, Department of Philosophy, Politics & Economics, 58448 Witten, Germany

**Keywords:** Politics, Government, Sociology

## Abstract

We describe the Archive of Interwar Europe Election Data and Assemblies (AIEEDA), a new multi-level dataset of parliamentary elections in interwar Europe (1919-1939). The data contains electoral results for all parties that ran in 137 national parliamentary elections in 25 interwar European democracies. It further offers detailed time-invariant ideological and organizational variables for 401 parliamentary parties and 35 party alliances, along with time-variant data on their participation in 412 cabinets. Next to national-level election results, we provide disaggregated constituency/municipality-level results for Estonia, Ireland, Italy, Latvia, the Netherlands, and Yugoslavia. Having collected national and disaggregated data independently, we validated each through the other. We also provide linking tables to parliamentary election results at the constituency/municipality-level for France, Germany, and the United Kingdom. The archive will be useful to social scientists interested in testing theories on voting and party politics out of sample in a historical setting, or to use historical cases to understand contemporary phenomena such as the rise of radical right-wing parties.

## Background & Summary

Both public and scholarly interest in the political dynamics of interwar democracies has increased considerably since the mid-2010s due to the perceived historical analogies in the rise of radical political actors in Europe and the United States^1,2^. Yet systematic research on fundamental democratic dynamics, such as electoral behavior, party system development, and government formation is hampered by the lack of encompassing, integrated, and reliable data on these variables from the interwar period. We provide such data by introducing the Archive of Interwar Europe Election Data and Assemblies (AIEEDA)^3^. The new data archive contains national election results (vote shares) for 996 parties, alliances, and independents that ran in 137 national parliamentary elections in 25 European democracies between January 1st, 1919 and August 31st, 1939. Additionally, AIEEDA offers detailed time-invariant ideological and organizational variables for 401 parliamentary parties and 35 party alliances with seats in parliament, along with time-variant data on their participation in 412 cabinets. Moreover, we provide geo-coded constituency/municipality-level election results for Estonia, Ireland, Italy, Latvia, the Netherlands, and Yugoslavia. AIEEDA data can be downloaded via a dedicated Open Science Framework (OSF) dataverse: 10.17605/OSF.IO/QS3DG.

**Fig. 1:**
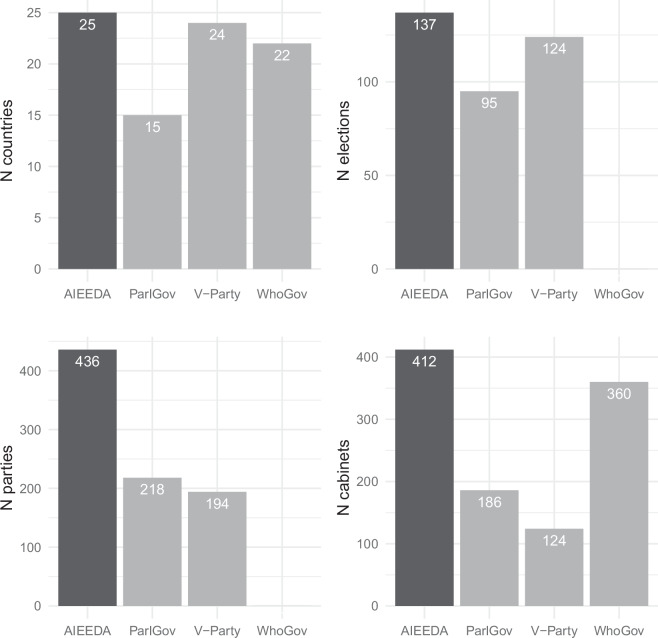
Coverage of AIEEDA and alternative data sources of countries, elections, parliamentary parties (including alliances), and cabinets for interwar European democracies.

AIEEDA goes beyond existing data compilations by providing greater coverage, and by integrating information across units (elections, parties, and cabinets) and levels of analysis (national and constituency/municipality-level results). Compared to the widely-used *ParlGov* data^4,5^, AIEEDA adds 42 elections (AIEEDA has 1.44 times as many elections as the *ParlGov* data), 220 parliamentary parties (2.01 times), 226 cabinets (2.22 times), and 10 new interwar democracies (1.67 times), nine of which would ultimately experience democratic breakdown during the interwar period. Since 12 of the 25 democracies in our sample do not survive until 1939, AIEEDA rectifies an imbalance between cases of democratic survival and breakdown in the *ParlGov* data. Relative to the popular *V-Party* dataset^6^, our data feature 242 additional parties including their first ideological classifications. AIEEDA expands coverage over the *Who Governs Europe* database^7^ by three countries and 52 cabinets (see Fig. 1 for a comparison between all datasets on all dimensions). Finally, it adds geo-coded constituency/municipality-level election results for five new interwar democracies, extending existing databases such as *CLEA*^8^ substantially, either by providing completely new data or by providing data at an additional level.

Beyond our research interests on the relationship between political violence and the success of anti-system parties, AIEEDA will be a valuable resource for social scientists, e.g., scholars who situate themselves in the emerging field of Historical Political Economy (HPE) that “spans not only the traditional subfields of political science but also economics, history, and sociology” [^9^,p.176]. Scholars working in this tradition have used disaggregated electoral results to investigate different relationships, including the effect of election results on anti-semitic violence in Poland^10^ and on targeting of political opponents during the Spanish Civil War^11^, the effect of veterans on electoral support for radical-right parties in France, Germany, and Italy^12–15^, or consequences of religious affiliation, political speeches, and radio propaganda on electoral support for the Nazis in Germany^16–18^.

The prevailing practice by HPE scholars to study large states mirrors a wider trend in political science to conduct single-country studies^9,19^. Our constituency/municipality-level electoral data allows investigation of previously understudied cases including Estonia, Latvia, the Netherlands, and Yugoslavia. AIEEDA’s integration of information on different levels of analysis allows scholars interested in disaggregated electoral dynamics to draw on national-level variables such as party’s ideological affiliations or their incumbency status. The broad, comparative focus of AIEEDA, moreover, permits research in a cross-national, comparative framework to study topics which have almost exclusively been investigated in post-Word War II (Western) Europe, such as government formation and durability^20,21^, party systems^22^, party polarization^23^, and fragmentation^24^.

## Methods

We begin by defining the universe of cases that are included in our dataset: European democracies in the interwar period. We selected all states identified as democratic by the Boix, Miller, and Rosato (BMR) political regimes dataset^25^. The BMR data classifies a state as democratic as of December 31st of given year if (1) its executive is directly or indirectly elected and responsible to either voters or a legislature, (2) free and fair elections determine the composition of the legislature, and (3) a majority of adult men has the right to vote [^25^,1530]. We adopted this simple definition of democratic regimes because it both maximizes coverage of cases during the interwar period and enables a reliable classification. Overall, our sample consists of 25 democratic states that were democratic for any length of time in the period January 1st, 1919 to August 31st 1939, the day before the beginning of World War II. Out of the 25 democracies, only 13 cases survived the entire period whereas 12 democracies collapsed, with Italy being the first case of breakdown in March 1922 and Spain the last in April 1939.

### National-Level Election & Cabinet Data

AIEEDA provides information on all national, lower-house parliamentary elections and cabinets in the sample period. We obtained election results and cabinet membership from a wide variety of sources. With respect to election data, we drew on a wealth of non-digitized election almanacs^26–28^, official records from election commissions or statistical offices, historical case studies^29^, or period-specific edited volumes^30^. We cross-referenced these sources and included those election results with the highest agreement across sources.

Multiple countries from Western Europe were already covered by the widely-used *ParlGov* dataset^4^, which provides election results and the partisan composition of cabinets. In those cases, we frequently built on information from *ParlGov*. We, however, changed the *ParlGov* election results by supplementing missing parties and disaggregating the “Other” or “Independent” categories whenever possible. We also added vote shares for a large number of electoral parties, which did not eventually enter parliament. We did not use *ParlGov* data when a majority of alternative sources indicated different election results.

Regarding cabinet-level data, we classify a new cabinet (i) whenever the prime minister changes, (ii) whenever the distribution of seats for a government party changes in parliament (e.g., through party splits or mergers and through elections), or (iii) when the combination of parties in the cabinet changes (e.g., through the inclusion of a new party). We do not code reshuffles of ministers between portfolios. We derived lots of information from the *Who Governs Europe* database^7^ for eight of the 11 cases not included in *ParlGov*. We again validated these classifications drawing on country-specific sources where possible. However, we differ from both *ParlGov* and *Who Governs Europe* in classifying new cabinets when the distribution of seats for government party changes. We argue that such a reconfiguration of bargaining power between government parties constitutes a new cabinet, even if cabinet membership remains the same. For the three countries missing from *Who Governs Europe*, Iceland, Italy, and Lithuania, we collected original data from case-specific sources. For each country, we provide a detailed list of references and coding notes on request.

### National-Level Party Data

For each parliamentary party in our data, we collected information on a range of variables from party ideology to social constituencies and organizational features (see Table 1 for party-level variables). Unlike existing efforts that survey experts to classify the ideological orientation of contemporary political parties at different points in time^31,32^, we based our assessment on historical case descriptions and provide a one-time snapshot. The time-invariant nature of our data is defensible in light of three observations: First, party competition in interwar Europe was clearly defined by class and nationalist conflict^33^, and most established party systems had been frozen by earlier social conflicts^34^. Second, among the more fluid party systems in younger democracies, frequent party splits and deaths mean that most classifications are up-to-date for any given election because we coded each new party that ran. Third, political parties during the interwar period did not publish election manifestos as they do today. Instead, many followed stable ideological principles, such as the German Catholic Center Party which only published one party program in 1922 that lasted throughout the entire interwar period.

**Table 1:** Summary Statistics of AIEEDA Party-Level Variables.

Statistic	N	Mean	St. Dev.	Min	Max
Econ. Left-Right	410	2.915	1.151	1	5
Religious Claim	446	0.240	0.428	0	1
Linguistic Claim	445	0.146	0.354	0	1
Majoritarian Nationalist	450	0.187	0.390	0	1
Territorial Claim	449	0.238	0.554	0	2
Rural Claim	457	0.217	0.412	0	1
Anti-System	447	0.215	0.411	0	1
Violent Wing	426	0.174	0.379	0	1
No. of Factions	506	2.028	4.215	0	58

To classify our party variables, we primarily translated historical case studies of parties, party families, and party systems^26,33,35–45^ into quantitative classifications. The wealth of political-historical studies provided information even about minor parties. Moreover, our international research team was able to access source material in the original languages spoken in 18 out of 25 states. In several cases, we furthermore contacted experts on interwar party competition to gain insights on parties for which we could not find reliable information.

To ensure the cross-case validity and reliability of our data, we proceeded in seven steps:

1. The core team of researchers (L.K., B.D.S., S.S., and N.-C.B.) prepared a codebook including the relevant conceptual definitions and operational criteria for each variable.
2. We adopted simple categorical or ordinal scales, thereby trading fine-grained differentiation for greater reliability. Similarly simple, contemporary approaches to party classification achieve broadly comparable results to more detailed expert surveys^46,47^.
3. We classified five parties, compared the results, and went back to the codebook to remove ambiguities that became clear from disagreements between our classifications (the Codebook includes definitions and a classification example).
4. We programmed an interactive Shiny app for data entry. The app separated data entry for each party and each variable into separate screens and displayed the conceptual and operational definition of each respective variable so that researchers were aware of it during data entry. Moreover, the app minimized data entry errors by only allowing the range of permissible values (including a “Do not know” option) for each variable (selected app screens can be found in the Supplementary Information).
5. We trained all members of the research team, including student research assistants and international colleagues, on the basis of the codebook, the party examples, and the Shiny app.
6. Prior to starting their main coding tasks, all team members had to classify a pre-defined set of ten parties that the core team had jointly classified. Each team member received feedback on their classifications.
7. Throughout the full classification process an online chat room allowed team members to communicate with one another to discuss borderline cases and the validity of sources.
8. Cases that proved difficult to classify were discussed by the core research team that took the ultimate decisions.

### Disaggregated Election Data

The final element of AIEEDA is a set of geo-coded, disaggregated national-level election results at the constituency or municipality-level from six different countries: Estonia, Ireland, Italy, Latvia, the Netherlands, and Yugoslavia. These disaggregated election results are connected to the national-level party and election data via joint ID variables. Moreover, we provide link files to previously published disaggregated national-level election results at different levels for France (municipality), Germany (county), and the United Kingdom (constituency)^8,48,49^. Table 2 provides an overview of the (linked) data by country including the unit of analysis, the number of elections, the number of units, and whether the data contain information on vote shares and/or seats.

**Table 2:** Overview of National Parliamentary Election Results at Constituency-/Municipality-Level.

Country	Electoral Rules	Unit Type	N Elections	N Units	Vote Shares	Seats	Source
Estonia	PR	Municipality	4	409–411	Yes	No	National Digital Archive^57^
Ireland	STV	Constituency	8	28–34	Yes	Yes	Walker^50^
Italy	PR	Constituency	2	54–40	Yes	No	General Directorate of Statistics^53^
Latvia	PR	Municipality	4	578–580	Yes	No	Central Electoral Commission^56^
Netherlands	PR	Municipality	5	852–1,074	Yes	No	Electoral Council^51^
Yugoslavia	PR	County	4	341-350	Yes	No	National Assembly^60^
**Links to other Data**							
France	Majoritarian & Mixed	Municipality	5	35,325	Yes	No	Cagé & Piketty^48^
Germany	PR	County	9	946	Yes	No	Falter & Haenisch^49^
United Kingdom	Plurality	Constituency	6	595–596	Yes	Yes	Kollman *et al*.^8^

**Fig. 2:**
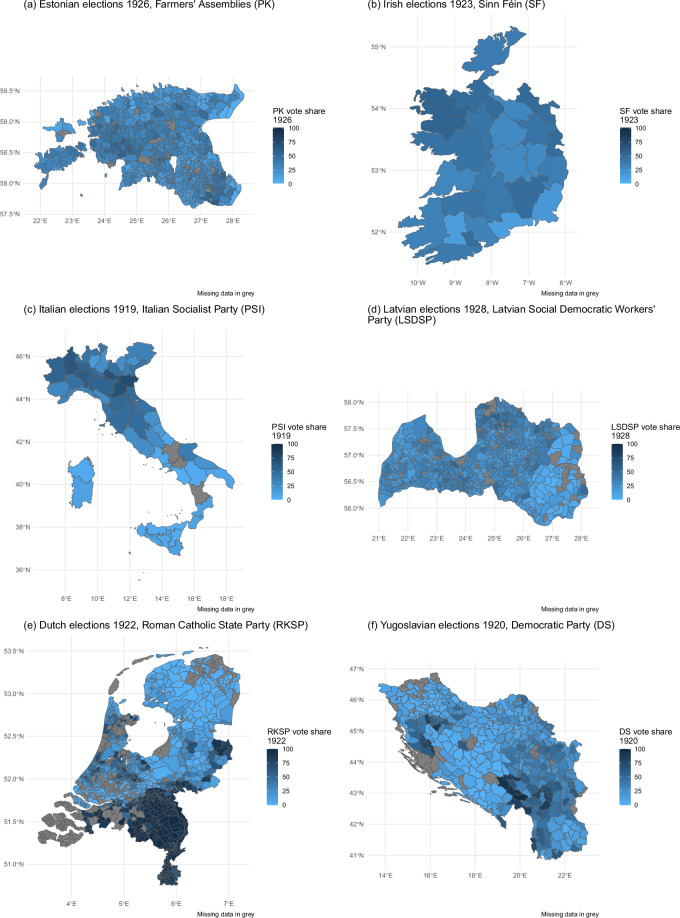
Local variation in vote shares of the strongest party in each election. Missing values are depicted in gray, water bodies in white.

We retrieved constituency/municipality-level election data from a variety of governmental and archival sources. For Ireland, we drew constituency-level election data and constituency boundaries from Walker^50^. We collected Dutch municipality-level election results and historical shapefiles from the Dutch Electoral Council and the Netherlands Geographic Information System project^51,52^. We obtained Italian election data from records kept by the Ministry of Economic Affairs, Directorate General of Statistics^53^, while we reconstructed constituency boundaries by assigning contemporary municipality shapefiles to historical constituencies^54,55^. For Latvia, we collected disaggregated electoral results from the official statistical records published by the Central Electoral Commission^56^. We acquired Estonian election data through official records from the National Digital Archive^57^. To geocode disaggregated units, we utilized Estonian and Latvian historical maps provided by the national libraries of both countries^58,59^. For Yugoslavia, we relied on historical parliamentary records^60^. As for unit boundaries, we employed county-level shapefiles based on the data from the 1931 census^61^. We geocoded disaggregated units for Estonia, Ireland, and Latvia using QGIS, a free and open-source geographic information system. Figure 2 depicts the largest party’s vote share by constituency/municipality for one exemplary democratic election of each country in the period 1919-1939.

We end this section by describing one difficulty that we encountered in several countries (Belgium, France, and Italy): the complexity of mapping constituency election results onto the final seat distribution in parliament due to the mismatch between electoral lists and parliamentary groups. We illustrate this problem with examples drawn from Italy. The so-called “Liberal Party” – a group of politicians with a shared ideological background, not formalized as a party until late 1922 – ran multiple lists, often centered around personalist factions. The different lists regrouped into different parliamentary party groups that did not align with the original electoral lists. For example, in 1921, three members elected from the *Unione Nazionale* list in Catania joined seven of the eleven members elected from the *Democratica Sociale* list to form one parliamentary group. In some cases, lists only served an electoral purpose and dissolved after the elections. For example, the five candidates elected from the already mentioned *Unione Nazionale* list joined three different parliamentary groups. This mismatch between electoral results at the constituency/municipality and the national-level is a fundamental feature of these early party systems that users of the data must consider when comparing electoral results at different levels.

## Data Records

We published all AIEEDA data together with the Codebook and the R code that recreates the Tables and Figures of this publication with the OSF^3^. The replication zip archive contains five folders (see Table 3 for an overview of the folder structure). The *code* folder contains the R code for replicating the Figures and Tables shown in this manuscript. The *data* folder contains all relevant data. Cabinet-level, election-level, and party-level data are made available as csv files. Moreover, we provide a separate file with the start and end dates of each country’s democratic period. Disaggregated election-level data are provided in separate sub-folders for each country. For Estonia, Ireland, Italy, Latvia, the Netherlands, and Yugoslavia, we made available shapefiles in the ESRI format along with the disaggregated election results. In the case of Yugoslavia, we provide county identifiers that are needed to link shapefiles and county-level election results. For France, Germany, and the UK, we provide linkage keys to disaggregated election results published by other researchers^8,48,49^ but not the original constituency/municipality-level election results. The *docs* folder contains the codebook, which explains the units of analysis and the classification of each variable in detail. The codebook also presents an example classification of one party. This folder also contains the Supplementary Information. The folders *figs* and *tables* contain the figures and tables which we created for this manuscript. Country notes and source files for each country are available upon request. Given our debt to the creators of the *ParlGov*^4^ and *Who Governs Europe*^7^ databases, we urge all users who work with our national-level election or cabinet data to cite these two data sources along with our data.

**Table 3:** Folder structure when downloading the AIIEDA data.

Folder	File	Content
code	counts-aieeda_data-250314.R	Count entities in AIEEDA data
	counts-from-parlgov-250314.R	Counts entities in *ParlGov* data^5^
	counts-from-vparty-250314.R	Counts entities from *V-Party* data^6^
	describe-aieeda-party_data-250314.R	Creates Table 1 and Figures 3, 4, and 5
	map-aieeda-fig2-elecresults-250314.R	Creates Figure 2
	plot-aieeda-fig1-comparedata-250314.R	Creates Figure 1
	plot-aieeda-fig3-scattter-lr_parfam-250314.R	Is run in the describe-aieeda-party_data code
	plot-aieeda-fig4-bar-partyfam_issue-250314.R	Is run in the describe-aieeda-party_data code
	plot-aieeda-fig5-bar-parfam_parorga-250314.R	Is run in the describe-aieeda-party_data code
	tab1-aieeda-party_summstats-250314.R	Is run in the describe-aieeda-party_data code
	tab4-aieeda-validation-250314	Creates Table 4
data	AIEEDA-cabinets-v1.csv	Cabinet-level data for 25 countries
	AIEEDA-elections-v1.csv	Election-level data for 25 countries
	AIEEDA-parties-v1.csv	Party-level data
	AIEEDA-start-end-dates-v1.csv	Start and end dates for countries’ democratic periods
	parlgov-cabinet-v2024.csv	A copy of the *ParlGov* cabinet data^5^
	parlgov-elections-v2024.csv	A copy of the *ParlGov* election data^5^
	summary.csv	Dataset with party, cabinet, country, and election counts for Fig. 1
	V-Dem-CPD-Party-V2.csv	A copy of the *V-Party* data^6^
	EE/AIEEDA-Estonia-subnat-v1.csv	Municipality-level data for Estonia
	EE/shapefiles	A folder that contains the shapefiles for Estonia
	FRA/AIEEDA-France-linkTable-v1.csv	Linkage keys to external French municipality-level data^48^
	GER/AIEEDA-Germany-linkTable-v1.csv	Linkage keys to external German county-level data^49^
	IE/AIEEDA-Ireland-subnat-v1.csv	Constituency-level data for Ireland
	IE/shapefiles	A folder that contains the shapefiles for Ireland
	IT/AIEEDA-Italy-subnat-v1.csv	Constituency-level data for Italy
	IT/shapefiles	A folder that contains the shapefiles for Italy
	LV/AIEEDA-Latvia-subnat-v1.csv	Municipality-level data for Latvia
	LV/shapefiles	A folder that contains the shapefiles for Latvia
	NL/AIEEDA-Netherlands-subnat-v1.csv	Municipality-level data for the Netherlands
	NL/shapefiles	A folder that contains the shapefiles for the Netherlands
	UK/AIEEDA-UnitedKingdom-linkTable-v1.csv	Linkage keys to external UK constituency-level data^8^
	YUG/AIEEDA-Yugoslavia-subnat-v1.csv	County-level data for Yugoslavia
	YUG/shapefiles-county-identifier.csv	Linkage keys for merging county maps to county election results
	YUG/shapefiles	A folder that contains the shapefiles for Yugoslavia
docs	AIEEDA-Codebook-v1.pdf	Describes units and variables, contains example coding for parties
figs	*multiple*	Contains all Figures from this manuscript
tables	*multiple*	Contains Tables 1 and 4

AIEEDA provides 3-digit identifier codes for countries, elections, cabinets, and parties. 3-digit country-level ID variables derive from the Correlates of War (*COW*) dataset^62^. 3-digit election, cabinet, and party-level IDs are unique to the AIEEDA data, and range between 100 and 999. Combining these ID variables, we provide 6-digit ID combinations of the *COW* country codes with election, cabinet, and party IDs that uniquely identify each unit across countries. Moreover, we provide election, cabinet, and party IDs from *ParlGov*^4^ and election IDs from the *Democratic Electoral Systems 1919-1945* data^63^ to facilitate cross-data comparisons.

## Technical Validation

We validate our election results by comparing national to constituency/municipality-level results. As described above, we obtained aggregated national-level data from secondary data sources, such as election almanachs. In contrast, our disaggregated data derives from predominantly primary sources published by country-specific statistical agencies or electoral commissions. Table 4 lists the 27 elections for which AIEEDA provides constituency/municipality-level data. It displays the mean deviation and root mean square error of party vote shares as captured by national and constituency/municipality-level data. The results of this validation exercise are encouraging. Only one election (Ireland, 1932) shows an average difference between national and disaggregated results of more than one percentage point. Four additional elections exhibit mean deviations of more than half a percentage point: two in Ireland and two in Yugoslavia. The remaining election results are nearly equivalent at the different levels. The larger deviations in Ireland stem from partially incomplete data at the constituency-level in the 1922 election and from independent candidates in the 1932 and 1933 elections. In Yugoslavia, independent candidates and lists fully explain the differences.

**Table 4:** Comparison of national and disaggregated national election results, deviations in percentage points (0-100).

Country	Election date	Avg. deviation	Root mean sq. err.
Estonia	May 1923	0.07	0.13
Estonia	May 1926	0.03	0.04
Estonia	May 1929	0.02	0.02
Estonia	May 1932	0.02	0.03
Ireland	June 1922	0.65	1.18
Ireland	August 1923	0.00	0.00
Ireland	June 1927	0.01	0.03
Ireland	September 1927	0.32	0.51
Ireland	February 1932	1.11	1.60
Ireland	January 1933	0.53	0.64
Ireland	July 1937	0.00	0.00
Ireland	June 1938	0.00	0.00
Italy	November 1919	0.14	0.23
Italy	May 1921	0.03	0.04
Latvia	October 1922	0.01	0.04
Latvia	October 1925	0.00	0.00
Latvia	October 1928	0.00	0.00
Latvia	October 1931	0.01	0.02
Netherlands	July 1922	0.07	0.17
Netherlands	July 1925	0.07	0.20
Netherlands	July 1929	0.00	0.00
Netherlands	April 1933	0.01	0.04
Netherlands	May 1937	0.00	0.00
Yugoslavia	November 1920	0.42	0.70
Yugoslavia	March 1923	0.53	0.93
Yugoslavia	February 1925	0.39	0.80
Yugoslavia	September 1927	0.58	0.91

While we provide election-level data at two levels that derive from distinct sources, we neither provide nor identify alternative sources for our party-level data, as we are the first to collect large-N information on party ideology in the interwar period. We therefore assess convergent and divergent inter-item validity. We anchor our comparison on the Party Family variable, which is central and explicit in the sources we used, and hardly varies over time. Political scientists share a common definition of how most party families align along a general left-right continuum^47,64^, and we order our party family classification accordingly with Communist and Socialist parties on the left, and Conservative and Fascist parties on the right. Contemporary ordering of party families does not include Agrarian parties. We place them on the center-left between Social Democratic and Christian Democratic parties, because most of them demanded redistributive land reform, even if their social values tended to be more conservative.

**Fig. 3:**
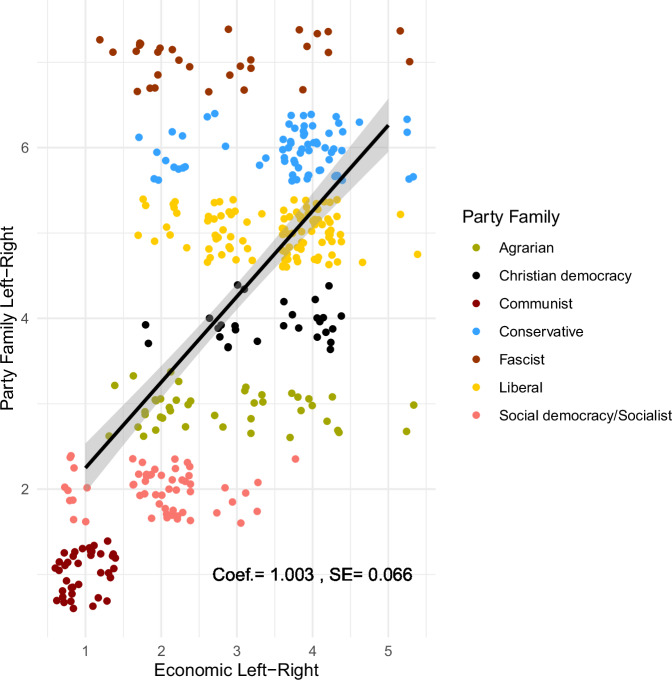
Scatter plot of economic left-right and party family variables. All values jittered to increase legibility. Party family order follows Kayser et al.’s^47^ classification (ethnic parties omitted). Solid black line displays bivariate correlation coefficient with 95% confidence intervals (shaded area).

We compare our classification of the five-point Economic Left-Right variable (horizontal axis) to the generalized party family left-right continuum (vertical axis) in Fig. 3. We find high agreement between the two classifications. Communist and Socialist/Social Democratic parties appear in the bottom left corner, whereas Liberal and Conservative parties predominantly show up in the top right. Agrarian and Christian Democratic parties scatter around the political spectrum with a center-left tilt in the case of the former and a center-right tilt for the latter. Fascist parties, which we consider to occupy the extreme right of a generalized left-right dimension, scatter across the economic left-right continuum, because they frequently advocated “national-socialist” policies. Overall, the statistical fit between the two variables is strong with a beta coefficient from a bi-variate linear model of 1.003 (*p* < 0.01).

**Fig. 4:**
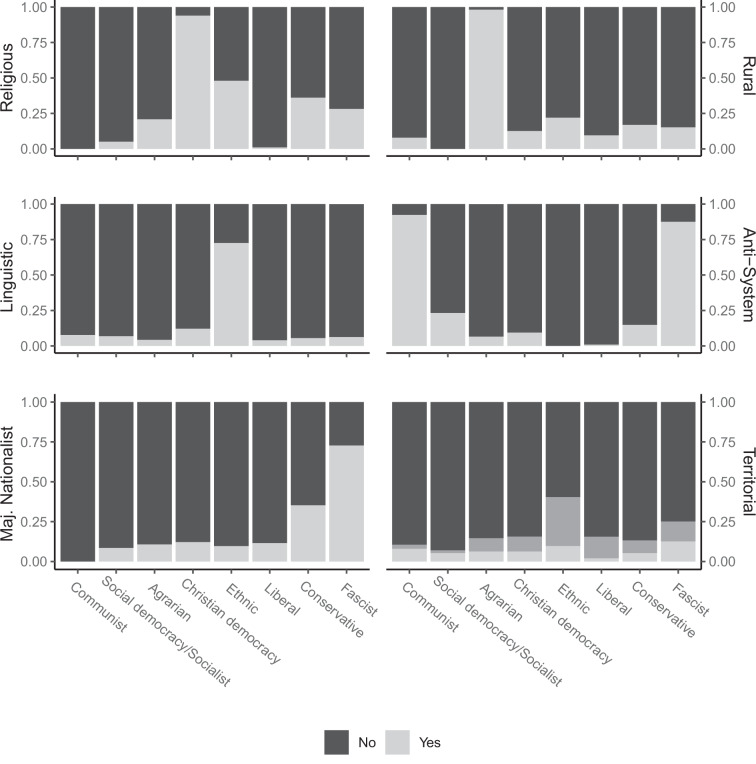
Marginal distributions of party ideological claims by party family. Legend in bottom right plot (Territorial claims): black = no territorial demands; dark grey = decentralization demands; light grey = secessionist demands.

Next, we contrast the party family classification used above with six distinct political claims that parties are advancing. Figure 4 plots the share of parties that advance religious, linguistic, majoritarian nationalist, rural, anti-system, and territorial claims by party family. We followed Lipset and Rokkan’s influential typology of social/political cleavages in Europe that identifies parties across four divisions: class (economic left-right), center-periphery, urban-rural, and church-state^34^. We added the more generic anti-system dimension to capture the interwar periods’ deep divisions over the appropriate system of government. Variable definitions and coding instructions are available in the Codebook. For all but the territorial dimension, the light gray share of the bar indicates that a claim is present. For territorial claims (bottom right), light gray indicates no claim, dark gray captures demands for autonomy, and black captures secessionist ambitions.

Christian democratic parties are the most vocal advocates of *religious issues*, whereas liberal, socialist, and communist parties hardly ever advance demands for a greater role of the sacred in public life. Unsurprisingly, almost all agrarian parties promote *rural demands*, while communist, socialist, and liberal parties with more urban voter bases hardly ever do so. Demands for greater *linguistic self-determination* in schools or public administration are almost exclusively found among ethnic parties. Communists and Fascists account for the vast majority of *anti-system* claims, although a minority of socialist and conservative parties does so as well. The frequency of parties supporting *majoritarian nationalism* increases as one moves from the generalized left to the right in the party spectrum, with the exception of ethnic parties. Finally, *territorial demands* for more decentralization or even independence are particularly pronounced among ethnic minority and fascist parties. Overall, party claims discriminate well between party families, indicating strong inter-item validity.

**Fig. 5:**
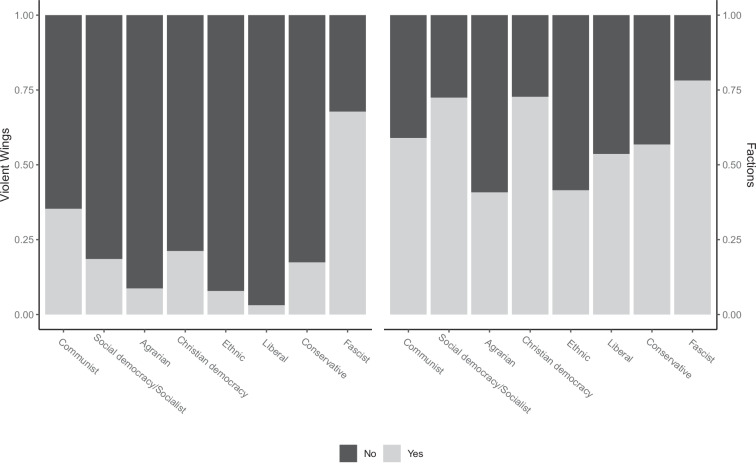
Marginal distributions of party organizational features by party family.

We conclude the validation section with a discussion of party family’s organizational characteristics. AIEEDA captures two organizational features: (1) whether or not parties feature *violent wings*, and (2) the number of internal *party factions*. Figure 5 displays marginal distribution of those two variables by party family. As expected, extreme parties on both sides of the spectrum had violent wings far more frequently than parties from the political center. Two-thirds of fascist parties in our data feature a violent wing, as do one-third of communist parties. Centrist party families have violent wings in no more than 22 out of 100 cases, with the highest share among Christian democrats followed by social democrats and conservatives. Turning to party factions, we report the share of divided parties and those without factions, rather than the number of factions as recorded in AIEEDA. While our data exhibits substantial variation across party families, no clear pattern is discernible. About three-quarters of socialist/social democratic, Christian democratic and fascist parties feature factions, and only around 50% of the remaining party families are internally divided. In sum, we find that the different party-level variables in our data converge and diverge relative to the Party Family variable in line with expectations, thus confirming the validity of the classification in the absence of an external gold standard.

## Supplementary information

Supplementary Information

## Data Availability

All code used to visualize the data is available together with the data on the OSF AIEEDA repository: 10.17605/OSF.IO/QS3DG.
