# Supplementary material for: Introducing the Archive of Interwar Europe Election Data & Assemblies (AIEEDA)
Source: Sci Data. 2025 Apr 15;12:630. doi: 10.1038/s41597-025-04969-y (PMC12000404; doi:10.1038/s41597-025-04969-y)
Supplement: Supplementary file 1 — Supplementary Information [file 41597_2025_4969_MOESM1_ESM.pdf]

A Supplementary Information

A.1 Shiny App

Coders were using a shiny app programmed for the purpose of coding parties. Figures A.1 through A.5 visualize the coding procedure.

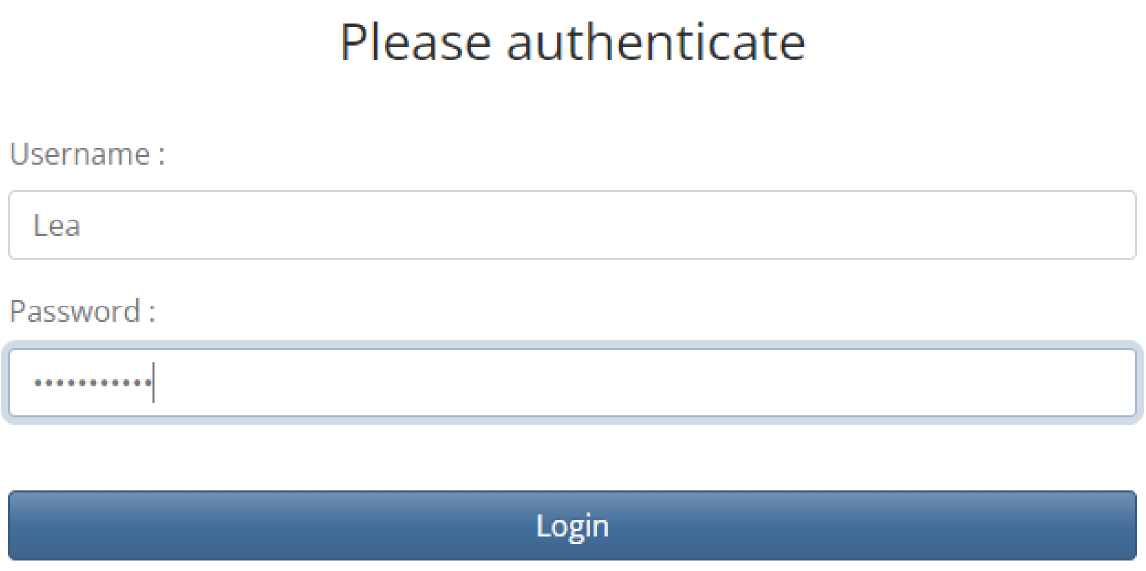

Figure A.1. Logging in to the shiny app avoids data entries from unauthorized sources.

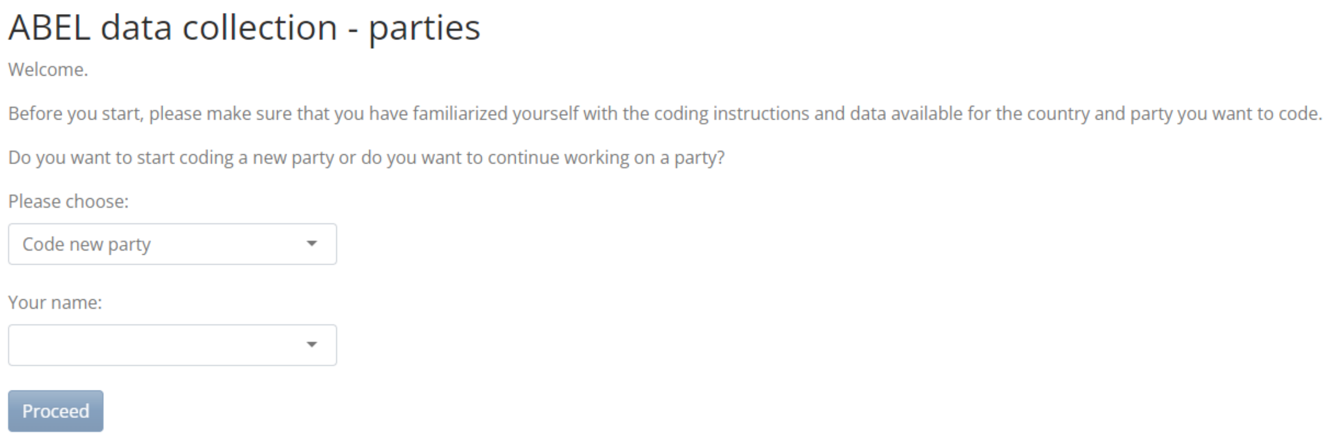

Figure A.2. After logging in, coders had to select whether to code a new party or continue coding a previous party.

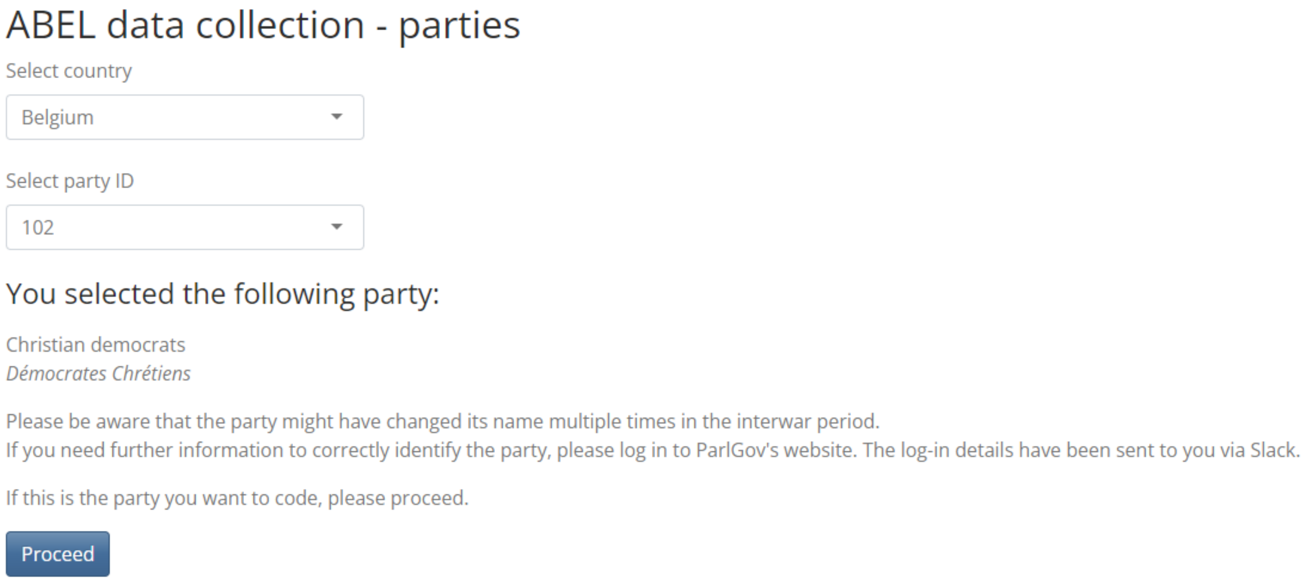

Figure A.3. Coders select the country and party they want to code. They can only proceed after having made valid choices.

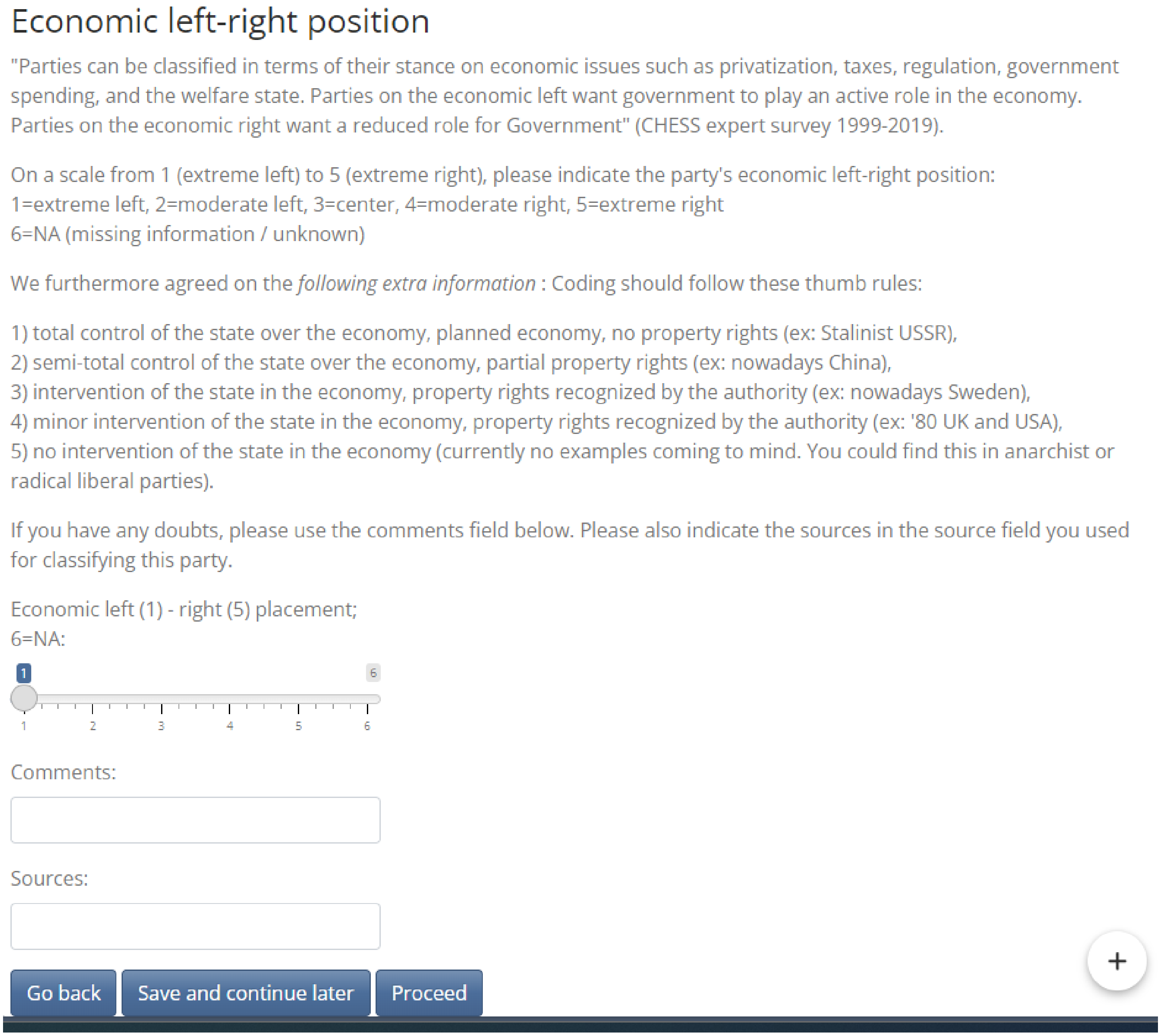
Economic left-right position

"Parties can be classified in terms of their stance on economic issues such as privatization, taxes, regulation, government spending, and the welfare state. Parties on the economic left want government to play an active role in the economy. Parties on the economic right want a reduced role for Government" (CHESS expert survey 1999-2019).

On a scale from 1 (extreme left) to 5 (extreme right), please indicate the party's economic left-right position:
1=extreme left, 2=moderate left, 3=center, 4=moderate right, 5=extreme right
6=NA (missing information / unknown)

We furthermore agreed on the *following extra information* : Coding should follow these thumb rules:

1) total control of the state over the economy, planned economy, no property rights (ex: Stalinist USSR),
2) semi-total control of the state over the economy, partial property rights (ex: nowadays China),
3) intervention of the state in the economy, property rights recognized by the authority (ex: nowadays Sweden),
4) minor intervention of the state in the economy, property rights recognized by the authority (ex: '80 UK and USA),
5) no intervention of the state in the economy (currently no examples coming to mind. You could find this in anarchist or radical liberal parties).

If you have any doubts, please use the comments field below. Please also indicate the sources in the source field you used for classifying this party.

Economic left (1) - right (5) placement;
6=NA:

1 6

1 2 3 4 5 6

Comments:

Sources:

Go back | Save and continue later | Proceed

+

Figure A.4. For each variable, coders were guided to a separate "page", being able to move back and forth, or stop coding and save their codings for later.

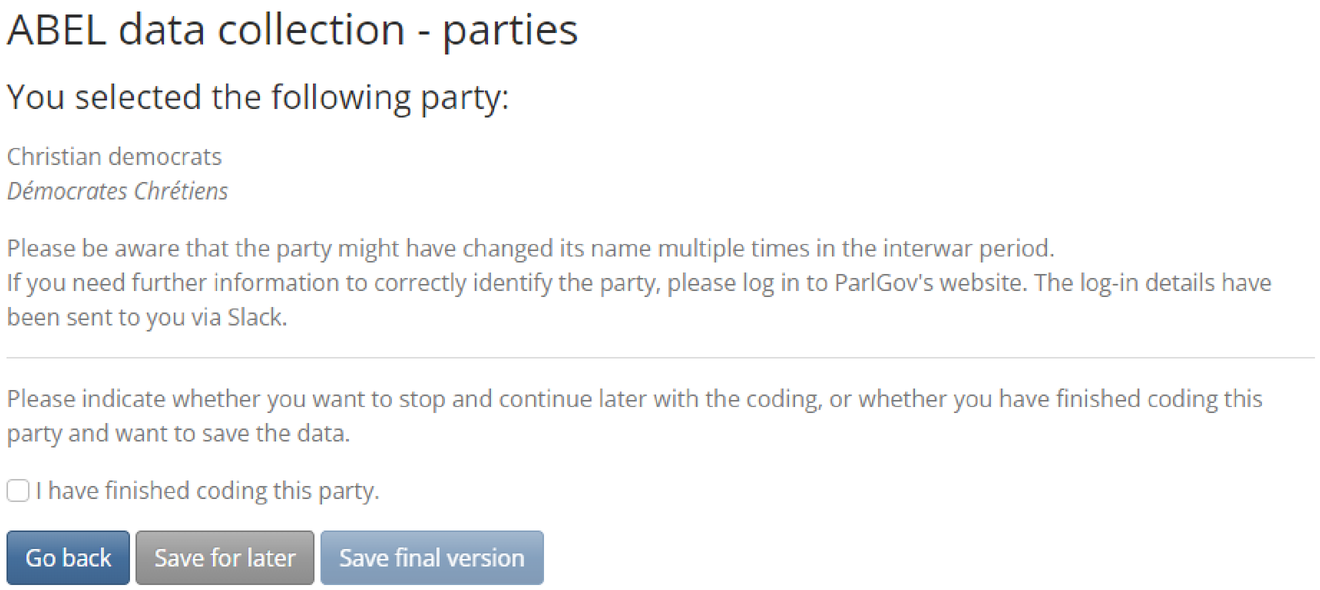

Figure A.5. On the final screen, coders could save their unfinished entries for later or indicate that they finished coding the party and save their final data.

Archive of Interwar Europe Election Data & Assemblies (AIEEDA)

Version: 1.0

Table of Content

Authors 1
Purpose 1
Data Units 2
Definitions of Key Terms 3
Variable descriptions 4
Election-level variables 4
Cabinet-level variables 11
Party-level variables 18
Election results at the sub-national level 27
Party Coding Example 30
References 35

Authors

Nils-Christian Bormann (Witten/Herdecke University)
Lea Kaftan (Leibniz Institute for the Social Sciences, Cologne)
Olga Jerjomina (Witten/Herdecke University)
Bruno della Sala (Witten/Herdecke University)
Stefan Stojkovic (Witten/Herdecke University)
Edoardo Viganò (Witten/Herdecke University)

Purpose

The Archive of Interwar Europe Election Data & Assemblies (AIEEDA) combines information on the actions and compositions of governments in Europe's interwar democracies (1919-1939). It identifies government and opposition parties, their ideological positions, their social basis, their parliamentary seats, and any actions governments take to weaken democratic institutions.

Data Units

At the most basic level the dataset includes political parties that gained parliamentary representation in Europe’s inter-war democracies. These parties are nested within election-cycles of countries identified as democracies by the Boix, Miller and Rosato (2013, 2018) dataset.

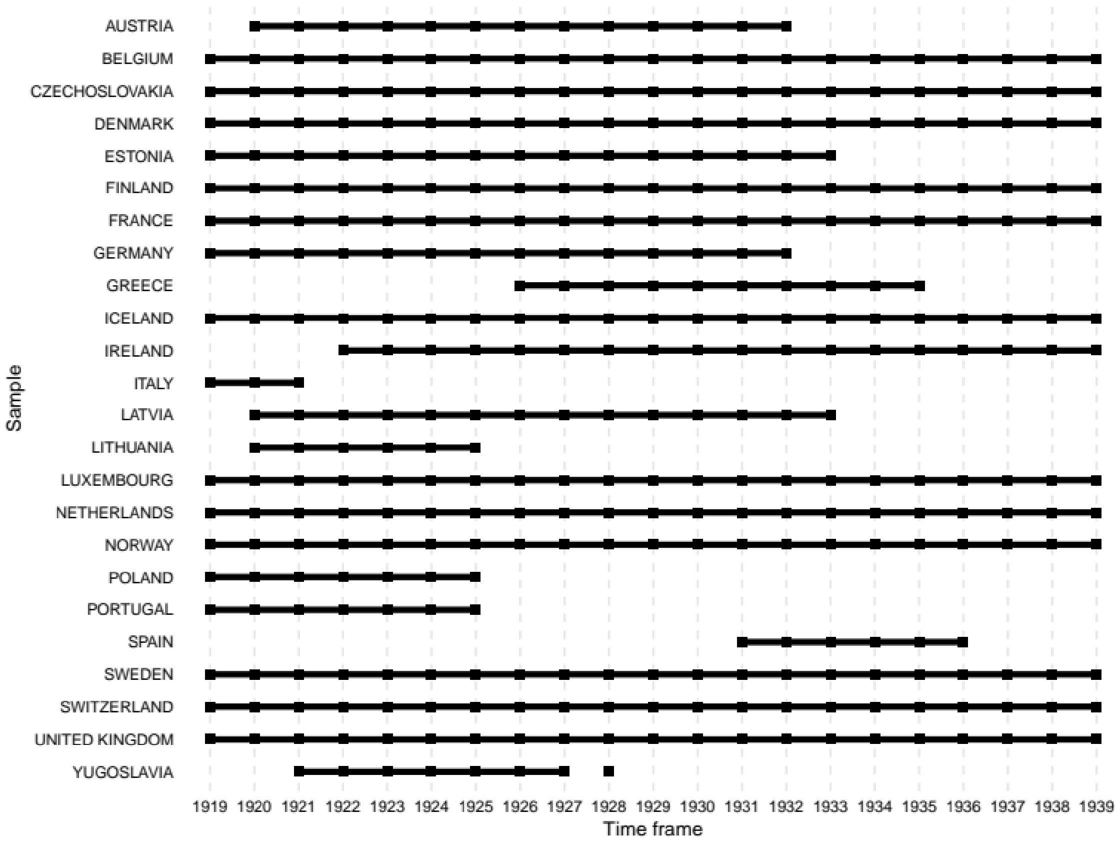

The dataset consists of three different levels of analysis:

1. The cabinet-level (AIEEDA-cabinets-v1.csv)
2. The party-level (AIEEDA-parties-v1.csv)
3. The election-level (AIEEDA-elections-v1.csv)

The cabinet-level includes all political parties that have won seats in a national-level parliament during the tenure of a given cabinet. It is time-variant and identifies the government participation and the number of parliamentary seats of parties since the last election.

The party-level includes each party only once. It is time-invariant and identifies support bases and other party characteristics that hardly change during the inter-war period.

The election-level includes information on votes and seats gained of each party in parliament for parties with at least 1 per cent of votes gained in the last election.

Definitions of Key Terms

Party

We include all parties mentioned in sources of electoral results in the election sheets, but only parties with at least one seat in parliament into the party and cabinet sheets. We mostly follow ParlGov's definition (Döring & Manow 2021).

In case there are no or only few official parties, we code electoral alliances (joined lists of candidates) for elections and parliamentary groups for cabinets and parties (see for example Italy in 1919). Whenever possible, we note down in the country notes how parties and electoral alliances relate to each other, and code their positions as weighted means of the positions of members. If only independents participated and if we cannot group them into electoral alliances or parliamentary groups, we do not code the election.

Cabinet

Cabinets are comprised of ministers. We code a government party, when a member of this party is a minister in the cabinet. Ministers are allowed to attend cabinet meetings and to cast a vote before the cabinet (ParlGov, Döring & Manow 2021). We do not consider junior ministers (e.g. Chief Secretaries and Parliamentary Under Secretaries of State in the UK). We code a cabinet change whenever the prime minister changes, the distribution of seats for a government party changes in parliament (e.g. through party splits or mergers and through elections) or when the combination of parties in the cabinet changes (e.g. through the inclusion of a new party). We do not code reshuffles of ministers.

Election

We code all parliamentary elections and the democratic elections for constituent assemblies. We only code elections that took place in democratic periods following Boix, Miller and Rosato (2013, 2018). If elections were held at several days or in several rounds, we code the date of the last day of any election.

Missing values

The data contain two different types of missing values. A "true" missing value (NA) indicates that coders have not entered any data into the respective field. A value that is referencing a missing value, e.g., "Don't know" (antisys) or 99 (left_right), indicates that coders did not find any information to make a valid decision. See variable descriptions for details.

Variable descriptions

Election-level variables

country_name_short

Type	Character
Value Range	
Level	Country
Description	Abbreviation in three characters of each party name
Sources	

country_name

Type	Character
Value Range	
Level	Country
Description	Name of the country fully written out
Sources	

cowid

Type	Integer
Value Range	
Level	Country
Description	Unique country ID, three digits
Sources	Singer & Small 1994

pgid

Type	Integer
Value Range	
Level	Country
Description	Unique country ID
Sources	Döring & Manow 2021

election_id_long

Type	Integer
Value Range	
Level	Election
Description	Unique election ID across countries, a combination of the cowid and the election_id
Sources	

election_id

Type	Integer
Value Range	
Level	Election
Description	Unique election ID within countries
Sources	

des_id

Type	Character
Value Range	
Level	Election
Description	Unique election ID across countries from the DES 1919-1949 data
Sources	Bormann & Kaftan 2024

election_date

Type	Date
Value Range	[1919-01-19; 1939-07-01]
Level	Election
Description	Day of the election; in case of several days: last day of the election
Sources	Döring & Manow 2021, Nohlen & Stöver 2010

election_type

Type	Character
Value Range	[“by-election”, “constituent assembly”, “parliament”]
Level	Election
Description	Type of the election, we have only covered parliamentary elections in all countries, by-elections and constituent assemblies only for some countries
Sources	Döring & Manow 2021, Nohlen & Stöver 2010, see also country notes and source files

party_id_long

Type	Integer
Value Range	
Level	Party
Description	Unique party ID across countries, a combination of cowid and party_id, six digits
Sources	

party_id

Type	Integer
Value Range	
Level	Party
Description	Unique party ID within countries
Sources	Döring & Manow 2021 and others (see country notes and source files)

party_name_short

Type	Character
Value Range	
Level	Party
Description	Party name abbreviation as in the source files. Be aware that these are not stable across elections.
Sources	Döring & Manow 2021 and others (see country notes and source files)

party_name

Type	Character
Value Range	
Level	Party
Description	Party name as in the source files in the original language, not stable across elections
Sources	Döring & Manow 2021 and others (see country notes and source files)

party_name_english

Type	Character
Value Range	
Level	Party
Description	Translation of the party name into English, not stable across elections
Sources	Döring & Manow 2021 and others (see country notes and source files)

vote_share

Type	Numeric
Value Range	
Level	Party
Description	Percentage of votes gained in the election
Sources	Döring & Manow 2021, Nohlen & Stöver 2010 and others (see country notes and source files)

seats

Type	Integer
Value Range	Z
Level	Party
Description	Number of seats in parliament
Sources	Döring & Manow 2021, Nohlen & Stöver 2010 and others (see country notes and source files)

parentid

Type	Integer
Value Range	
Level	Party
Description	Party ID of party that this party was previously part of<br>We do not code party alliances in which parties kept their organizational identity as former parents.
Sources	See country notes and source files

childid

Type	Integer
Value Range	
Level	Party
Description	Party ID of party/parties that split from this party<br>We only code first-generation descendants. For example, Germany’s Social Democrats split into Social Democrats (SPD) and Independent Social Democrats (USPD in 1917. The SPD would feature the party of the USPD in this variable but not the party id of the Communist Party of Germany (KPD), which split from the USPD in 1918.
Sources	See country notes and source files

party_type

Type	Character
Value Range	[“Alliance”, “Independents”, “Others”, NA]
Level	Party
Description	Specifies if the entity is rather an alliance, a single or a group of independents, or just specified as “others” in the original source. If nothing is specified here, we believe that the entity is best characterized as a party.
Sources	See country notes and sources files

alliance_members

Type	Character
Value Range	
Level	Party
Description	If the entity is an alliance, we list the IDs of the alliance members. Information on these members can be found in the AIEED-parties-v1.csv file.
Sources	See country notes and sources files

prev_election_id

Type	Integer
Value Range	
Level	Election
Description	election_id of the cabinet that preceded this cabinet, three digits, unique within countries, only
Sources	

prev_cabinet_id

Type	Integer
Value Range	
Level	Cabinet
Description	cabinet_id of the cabinet that preceded this cabinet, three digits, unique within countries only
Sources	

party_id_parlgov

Type	Integer
Value Range	
Level	Party
Description	Unique party ID in ParlGov
Sources	Döring & Manow 2021

election_id_parlgov

Type	Integer
Value Range	
Level	Cabinet
Description	Unique election ID in ParlGov
Sources	Döring & Manow 2021

prev_election_id_parlgov

Type	Integer
Value Range	
Level	Cabinet
Description	election_id_parlgov of the previous parliamentary election
Sources	Döring & Manow 2021

prev_cabinet_id_parlgov

Type	Integer
Value Range	
Level	Cabinet
Description	Unique cabinet ID in ParlGov of the cabinet preceding this cabinet
Sources	Döring & Manow 2021

Cabinet-level variables

country_name_short

Type	Character
Value Range	
Level	Country
Description	Abbreviation in three characters of each party name
Sources	

country_name

Type	Character
Value Range	
Level	Country
Description	Name of the country fully written out
Sources	

cowid

Type	Integer
Value Range	
Level	Country
Description	Unique country ID, three digits
Sources	Singer & Small 1994

pgid

Type	Integer
Value Range	
Level	Country
Description	Unique country ID
Sources	Döring & Manow 2021

cabinet_id_long

Type	Integer
Value Range	
Level	Cabinet
Description	Unique cabinet ID across countries, a combination of cowid and cabinet_id, six digits
Sources	

cabinet_id

Type	Integer
Value Range	
Level	Cabinet
Description	Unique cabinet ID within countries, three digits
Sources	

cabinet_name

Type	String
Value Range	
Level	Cabinet
Description	Name of prime minister and number of cabinet formed.
Sources	Casal Bértoa & Enyedi 2022, Döring & Manow 2021

caretaker

Type	Integer
Value Range	[0; 1]
Level	Cabinet
Description	1 if the cabinet has been a caretaker cabinet
Sources	Döring & Manow 2021 and others (see country notes and source files)

start_date

Type	Date
Value Range	[1919-01-27; 1939-08-10]
Level	Cabinet
Description	Day of the formation of the cabinet

Sources	Casal Bértoa & Enyedi 2022, Döring & Manow 2021

end_date

Type	Date
Value Range	[1919-06-21; 1939-08-31]
Level	Cabinet
Description	Day before the start of the next cabinet, or day in which the country became an authoritarian state
Sources	Casal Bértoa & Enyedi 2022, Döring & Manow 2021

party_id_long

Type	Integer
Value Range	
Level	Party
Description	Unique party ID across countries, a combination of cowid and party_id, six digits
Sources	

party_id

Type	Integer
Value Range	
Level	Party
Description	Unique party ID within countries
Sources	

party_name_short

Type	Character
Value Range	
Level	Party
Description	Translation of the party name into English, not stable across elections
Sources	Döring & Manow 2021, Nohlen & Stöver 2010 and others (see country notes and source files)

party_name

Type	Character
Value Range	
Level	Party
Description	Party name as in the source files in the original language, not stable across elections
Sources	Döring & Manow 2021, Nohlen & Stöver 2010 and others (see country notes and source files)

party_name_english

Type	Character
Value Range	
Level	Party
Description	Translation of the party name into English, not stable across elections
Sources	Döring & Manow 2021 and others (see country notes and source files)

seats

Type	Integer
Value Range	Z
Level	Party
Description	Number of seats in parliament.
Sources	Döring & Manow 2021, Nohlen & Stöver 2010 and others (see country notes and source files)

prime_minister

Type	Double Precision
Value Range	[0; 1]
Level	Party
Description	Binary flag whether the party leads the government by holding the office of prime minister, chancellor, head of government etc.
Sources	Casal Bértoa & Enyedi 2022, Döring & Manow 2021, Nohlen & Stöver 2010 and others (see country notes and source files)

cabinet_party

Type	Double Precision
Value Range	[0; 1]
Level	Party
Description	Binary flag whether party is included in government or not. We identify cabinet representation by ministerial portfolios. If a party holds any ministry, this variable takes the value of “1“. Otherwise code as “0”.
Sources	Casal Bértoa & Enyedi 2022, Döring & Manow 2021, Nohlen & Stöver 2010 and others (see country notes and source files)

election_date

Type	Date
Value Range	[1919-01-19; 1939-07-01]
Level	Election
Description	Day of the last election
Sources	Döring & Manow 2021, Nohlen & Stöver 2010

election_id

Type	Integer
Value Range	
Level	Election
Description	Unique election ID within countries
Sources	

des_id

Type	Character
Value Range	
Level	Election
Description	Unique election ID across countries from the DES 1919-1949 data
Sources	Bormann & Kaftan 2024

party_type

Type	Character
Value Range	[“Alliance”, “Independents”, “Others”, NA]
Level	Party
Description	Specifies if the entity is rather an alliance, a single or a group of independents, or just specified as “others” in the original source. If nothing is specified here, we believe that the entity is best characterized as a party.
Sources	See country notes and sources files

alliance_members

Type	Character
Value Range	
Level	Party
Description	If the entity is an alliance, we list the IDs of the alliance members. Information on these members can be found in the AIEEDA-parties-v1.csv file.
Sources	See country notes and sources files

entity_coded

Type	Integer
Value Range	[0; 1]
Level	Party
Description	If 1, we have information on the entity’s ideology available, otherwise not
Sources	

prev_cabinet_id

Type	Integer
Value Range	
Level	Cabinet
Description	Unique cabinet ID of the cabinet that preceded this cabinet, three digits, unique only within countries
Sources	

party_id_parlgov

Type	Integer
Value Range	
Level	Party
Description	Unique party ID in ParlGov
Sources	Döring & Manow 2021

cabinet_id_parlgov

Type	Integer
Value Range	
Level	Cabinet
Description	Unique cabinet ID in ParlGov
Sources	Döring & Manow 2021

election_id_parlgov

Type	Integer
Value Range	
Level	Cabinet
Description	Unique election ID in ParlGov
Sources	Döring & Manow 2021

prev_cabinet_id_parlgov

Type	Integer
Value Range	
Level	Cabinet
Description	Unique cabinet ID in ParlGov of the cabinet preceding this cabinet
Sources	Döring & Manow 2021

Party-level variables

country_name

Type	Character
Value Range	
Level	Country
Description	Name of the country fully written out
Sources	

cowid

Type	Integer
Value Range	
Level	Country
Description	Unique country ID, three digits
Sources	Singer & Small 1994

pgid

Type	Integer
Value Range	
Level	Country
Description	Unique country ID
Sources	Döring & Manow 2021

party_id_long

Type	Integer
Value Range	
Level	Party
Description	Unique party ID across countries, a combination of cowid and party_id, six digits
Sources	

party_id

Type	Integer
Value Range	
Level	Party
Description	Unique party ID within countries
Sources	Döring & Manow 2021 and others (see country notes and source files)

party_name_short

Type	Character
Value Range	
Level	Party
Description	Party name abbreviation as in the source files, not stable across elections
Sources	Döring & Manow 2021 and others (see country notes and source files)

party_name

Type	Character
Value Range	
Level	Party
Description	Party name as in the source files in the original language, not stable across elections
Sources	Döring & Manow 2021 and others (see country notes and source files)

party_name_english

Type	Character
Value Range	
Level	Party
Description	Translation of the party name into English, not stable across elections
Sources	Döring & Manow 2021 and others (see country notes and source files)

family_name

Type	Character
Value Range	[“Agrarian”, “Christian democracy”, “Communist”, “Conservative”, “Don’t know”, “Fascist”, “Liberal”, “Other”, “Social democracy/Socialist”, “Special Issue”, NA]
Level	Party
Description	Agrarian: Agrarian parties have a support base mainly comprised of small or large scale farmers and claim to support the interests of farmers.<br>Christian democracy: Christian parties have a support base mainly comprised of Christians, claim to represent Christian values and apply these values to economic and cultural policy making. They furthermore do not speak out against democratic rule.<br>Communist: Communist parties claim to support the interests of workers and lower class people. They campaign against representative democracy, although they might support forms of Soviet democracy.<br>Conservative: Conservative parties aim at preserving traditional institutions, values and hierarchies in their country. For the interwar period, this might include parties that demand a (partial) restoration of the monarchies or that defend the social, economic and political positions of the old elite.<br>Fascist: Fascist parties are openly anti-democratic and aim at putting into power a single leader. They share a nationalist homogeneous image of “the people”, are militaristic and believe that opponents to the leader are traitors who should be strongly punished.<br>Liberal: Liberal parties have a mainly urban support base, claim to represent modern secular values in opposition to traditional (Christian) values and / or demand only marginal involvement of the state and the Church in either economic or “private” cultural issues.<br>Social democracy/Socialist: Social democratic and socialist parties have an (extreme) left-wing political platform but support the ideal of representative democracy. They claim to represent the interests of the workers, lower class people and marginalized groups, and aim at reducing social and economic inequalities.<br>Special issue: Special issue parties only support the interest of a very specific and clearly defined part of the countries’ population or a narrow cause. One example is the Schleswig Party of Denmark.
Sources	See country notes and source files

left_right

Type	Integer
Value Range	[1-5, 99, NA]
Level	Party
Description	Economic left-right ordering of political parties (1=extreme left, 2=moderate left, 3=center, 4=moderate right, 5=extreme right).<br><br>We follow the CHESS expert survey’s classification: “Parties can be classified in terms of their stance on economic issues such as privatization, taxes, regulation, government spending, and the welfare state. Parties on the economic left want government to play an active role in the economy. Parties on the economic right want a reduced role for Government” (Bakker et al. 2020, 22).<br><br>For parties included in the ParlGov data base (Döring & Manow 2021), we recode the 11-point state-market scale and classify parties the following way: 0:1 as “extreme left”, 2:4 as “moderate left”, 5 as “centre”, etc.<br><br>We code missing information as 6=NA.<br><br><u>We provided the following additional coding instructions</u>:<br>1) total control of the state over the economy, planned economy, no property rights (ex: Stalinist USSR) 2) semi-total control of the state over the economy, partial property rights (ex: nowadays China) 3) intervention of the state in the economy, property rights recognized by the authority (ex: nowadays Sweden) 4) minor intervention of the state in the economy, property rights recognized by the authority (ex: '80 UK and USA) 5) no intervention of the state in the economy
Sources	See country notes and source files

rel_claim_flag

Type	Character
Value Range	[“Don’t know”, “No”, “Yes”, NA]
Level	Party
Description	Binary flag whether party claims to act on behalf of or attracts disproportional electoral support from a specific religious group
Sources	See country notes and source files

lin_claim_flag

Type	Character
Value Range	[“Don’t know”, “No”, “Yes”, NA]
Level	Party
Description	Binary flag whether party claims to act on behalf of or attracts disproportional electoral support from a specific linguistic group
Sources	See country notes and source files

eth_base_group

Type	Character
Value Range	
Level	Party
Description	Character value of party‘s linguistic or religious base<br>If there is no evidence that party represents a specific group, we code the country’s majority group.
Sources	See country notes and source files

majnat_claim

Type	Character
Value Range	[“Don’t know”, “No”, “Yes”, NA]
Level	Party
Description	Binary flag whether the party persistently, publicly and explicitly claims to represent the interests of the ethnic majority in the country or speaks out against proposed or existing rights of ethnic minorities
Sources	See country notes and source files

terr_claim

Type	Character
Value Range	[“Decentralization”, “Don’t know”, “No”, “Secession”, NA]
Level	Party
Description	Categorical variable whether party demands more autonomy for a specific region within the current nation state (“Decentralization”), demands secession of a specific region from the nation state (“Secession”) or makes no such claims (“No”)
Sources	See country notes and source files

rural_claim

Type	Character
Value Range	[“Don’t know”, “No”, “Yes”, NA]
Level	Party
Description	Binary flag whether party claims to act on behalf of or attracts disproportional electoral support from rural constituencies, i.e. farmers
Sources	See country notes and source files

antisys

Type	Character
Value Range	[“Don’t know”, “No”, “Yes”, NA]
Level	Party
Description	Binary flag whether party aims to implement a non-representative democratic system of government such as monarchy, fascist dictatorship, communist dictatorship, Soviet democracy, or other forms of political systems that are not representative democracies
Sources	See country notes and source files

violent_wing_flag

Type	Character
Value Range	[“Don’t know”, “No”, “Yes”, NA]
Level	Party
Description	Binary flag whether party has a violent wing, i.e., members of the party or an extra-party organization that answers to party leaders and commits acts of violence against political opponents or the state<br>Examples include the NSDAP’s Sturmabteilung (SA) or the Social Democrats “Reichsbanner Schwarz-Rot-Gold” in Germany.
Sources	See country notes and source files

violent_wing_name

Type	Character
Value Range	
Level	Party
Description	Name of violent wing
Sources	See country notes and source files

factions

Type	Character
Value Range	
Level	Party
Description	Character value of party factions (multiple factions possible)<br>Examples include the “Spartakusbund” in Germany’s Independent Social Democrats (USPD) that split off the USPD and formed the Communist Party of Germany in December 1918.
Sources	See country notes and source files

faction_count

Type	Integer
Value Range	
Level	Party
Description	The maximum number of factions that existed at the same time if there is some variation in the faction_count in the interwar period<br>Minimum coding for factions, if there are any: 2<br>If no factions, coded as 0
Sources	See country notes and source files

party_type

Type	Character
Value Range	[“Alliance”, “Independents”, “Others”, NA]
Level	Party
Description	Specifies if the entity is rather an alliance, a single or a group of independents, or just specified as “others” in the original source<br>If nothing is specified here, we believe that the entity is best characterized as a party.
Sources	See country notes and sources files

alliance_members

Type	Character
Value Range	
Level	Party
Description	If the entity is an alliance, we list the IDs of the alliance members. Information on these members can be found in the AIEEDA-parties-v1.csv file.
Sources	See country notes and sources files

party_id_parlgov

Type	Integer
Value Range	
Level	Party
Description	Unique party ID in ParlGov
Sources	Döring & Manow 2021

entity_coded

Type	Integer
Value Range	[0; 1]
Level	Party
Description	If 0, we have information on the entity’s ideology available. Otherwise not.
Sources	

coder_name

Type	Character
Value Range	
Level	Party
Description	Name of the coder, if “Aggregated”, we have calculated the position of the entity by aggregating the information of its constituent entities
Sources	

codingDate

Type	Date
Value Range	[2021-12-02; 2022-11-17]
Level	Party
Description	Name of the coder
Sources	

[var]_comment

Type	Character
Value Range	
Level	Party
Description	There is one comment column for each variable we coded. Comments are not cleaned.
Sources	

[var]_source

Type	Character
Value Range	
Level	Party
Description	There is one column for each variable we coded that lists the sources. Entries are not cleaned, yet, and refer to the source files for each country. Source numbers listed are only unique within countries.
Sources	

Election results at the sub-national level

Overview

We collected election results at the sub-national level data for Estonia, Ireland, Italy, Latvia, the Netherlands, and Yugoslavia. The sub-national level data can be merged with the national level data using the election_date, election_id, and party_id. It contains the following variables:

Var	Description
unit_name	Name of the territorial district the election results are listed for
unit_type	Type of the territorial district: either municipality
elec_district	Binary flag whether the district is also an electoral district
votes	Absolute number of votes won for each party in each district

We provide scripts for linking sub-national data with our data for France, Germany, and United Kingdom.

Estonia

File name: AIEEDA-Estonia-subnat-v1.csv

Shapefiles: available

Notes: For Estonia, we have digitized one map, so far, due to resource limits. Some regions in the map data are not covered by the sub-national election data. We attribute these differences to changes in boundaries. When researchers want to use the shapefile for Estonia, we encourage them to plot all regions covered in the shapefile, and not only the ones in the election data. The sub-national election data is still complete given that the results sum up to national level results.

Sources: DIGAR: https://www.digar.ee/viewer/et/nlib-digar:437257/371421/page/1

France

File name: AIEEDA-France-linkTable-v1.csv

Shapefiles: not available

Sources: Cagé and Piketty (2023)

Germany

File name: AIEEDA-Germany-linkTable-v1.csv

Shapefiles: not available

Sources: Falter and Hänisch (1990)

Ireland

File name: AIEEDA-Estonia-subnat-v1.csv

Shapefiles: available

Notes: Dublin is not drawn in the maps because the source does not provide enough detail to draw them accurately.

Sources: Walker (1992).

Italy

File name: AIEEDA-Italy-subnat-v1.csv

Shapefiles: available

Notes: We reconstructed constituency boundaries by assigning contemporary municipality shapefiles to historical districts.[1]

Sources: Statistica delle Elezioni Generali Politiche per la 26. Legislatura: 15 Maggio 1921 (Ministero dell'Economia Nazionale, Direzione Generale della Statistica: Roma, 1924).

Latvia

File name: AIEEDA-Latvia-subnat-v1.csv

Shapefiles: not available

Notes: For Latvia, we have digitized one map, so far, due to resource limits. Some regions in the map data are not covered by the sub-national election data. We attribute these differences to changes in boundaries. When researchers want to use the shapefile for Latvia, we encourage them to plot all regions covered in the shapefile, and not only the ones in the election data. The sub-national election data is still complete given that the results sum up to national level results.

Sources: https://kartes.lndb.lv/details/656166

Netherlands

File name: AIEEDA-Netherlands-subnat-v1.csv

Shapefiles: available

Notes: In some cases, election data might be missing for specific regions (see for example the Tweede Kamer 1922 results: (https://www.verkiezingsuitslagen.nl/verkiezingen/). Here, it specifies: "In 1922, CBS did not publish election statistics. The Electoral Council has collected results per municipality from municipal and provincial archives. There are many

[1] https://www.istat.it/notizia/confini-delle-unita-amministrative-a-fini-statistici-al-1-gennaio-2018-2/ and https://situas.istat.it/web/##/home

gaps. Comparison of the totals with the results per constituency in the official report indicates where those gaps are.” The election results aggregate well to the national level statistics which is why we believe that mismatches are minor and ignorable.

Sources: official statistics[2] and Netherlands Geographic Information System project[3]

United Kingdom

File name: AIEEDA-UnitedKingdom-linkTable-v1.csv

Shapefiles: not available

Sources: Kollman et al. (2019)

Yugoslavia

File name: AIEEDA-Yugoslavia-subnat-v1.csv

Shapefiles: available, including the shapefile-county-identifier.csv file for linking

Notes: The shapefiles contain information on all counties available, while the number of counties in which elections took place varies over the years. For example, elections could not take place in all 378 counties in 1920 because some were under Italian control or did not yet exist. We encourage users to plot all counties for the interwar period but provide election results only for those counties in which elections took place.

Sources: Popovic, M. 2020

[2] https://www.verkiezingsuitslagen.nl.

[3] https://https://nlgis.nl/.

Party Coding Example

Consider the German party *Bavarian Peasants‘ League* (BPL; partyid 2701). The sources in the Germany folder contained no useful references to the BPL. In contrast, a Wikipedia search led to a “stub” page (Figure 1).[4]

Figure 1: English Wikipedia Stub Page on the Bavarian Peasant's League. "Stub" disclaimer and German language link highlighted.

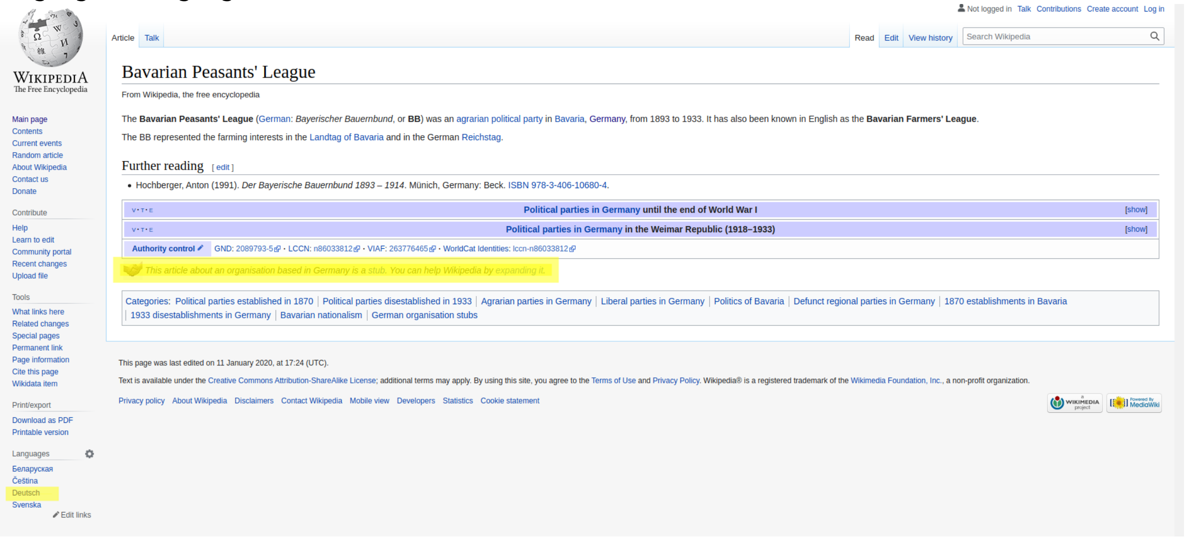

Not logged in Talk Contributions Create account Log in

Article Talk Read Edit View history Search Wikipedia

WIKIPEDIA The Free Encyclopedia

Main page
Contents
Current events
Random article
About Wikipedia
Contact us
Donate

Contribute
Help
Learn to edit
Community portal
Recent changes
Upload file

Tools
What links here
Related changes
Special pages
Permanent link
Page information
Cite this page
Wikidata item

Print/export
Download as PDF
Printable version

Languages
Беларуская
Čeština
Deutsch
Svenska
Edit links

Bavarian Peasants' League

From Wikipedia, the free encyclopedia

The **Bavarian Peasants' League** (German: *Bayerischer Bauernbund*, or **BB**) was an agrarian political party in Bavaria, Germany, from 1893 to 1933. It has also been known in English as the **Bavarian Farmers' League**.

The BB represented the farming interests in the Landtag of Bavaria and in the German Reichstag.

Further reading [edit]

- Hochberger, Anton (1991). *Der Bayerische Bauernbund 1893 – 1914*. Münich, Germany: Beck. ISBN 978-3-406-10680-4.

V • T • E Political parties in Germany until the end of World War I [show]

V • T • E Political parties in Germany in the Weimar Republic (1918–1933) [show]

Authority control GND: 2089793-5 • LCCN: n86033812 • VIAF: 263776465 • WorldCat Identities: lccn-n86033812

This article about an organisation based in Germany is a stub. You can help Wikipedia by expanding it.

Categories: Political parties established in 1870 | Political parties disestablished in 1933 | Agrarian parties in Germany | Liberal parties in Germany | Politics of Bavaria | Defunct regional parties in Germany | 1870 establishments in Bavaria | 1933 disestablishments in Germany | Bavarian nationalism | German organisation stubs

This page was last edited on 11 January 2020, at 17:24 (UTC).

Text is available under the Creative Commons Attribution-ShareAlike License; additional terms may apply. By using this site, you agree to the Terms of Use and Privacy Policy. Wikipedia® is a registered trademark of the Wikimedia Foundation, Inc., a non-profit organization.

Privacy policy About Wikipedia Disclaimers Contact Wikipedia Mobile view Developers Statistics Cookie statement

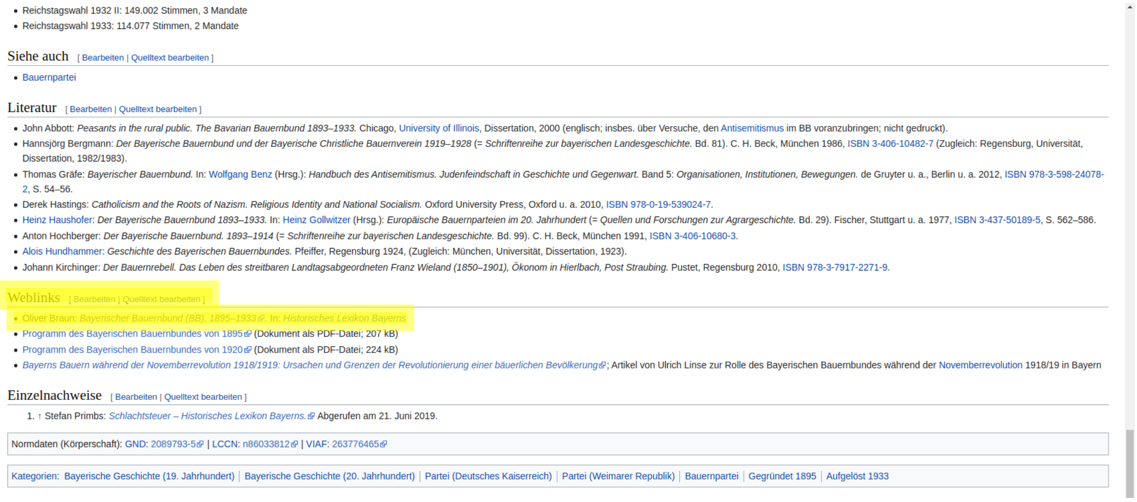

- Reichstagswahl 1932 II: 149.002 Stimmen, 3 Mandate
- Reichstagswahl 1933: 114.077 Stimmen, 2 Mandate

Siehe auch [Bearbeiten | Quelltext bearbeiten]

- Bauernpartei

Literatur [Bearbeiten | Quelltext bearbeiten]

- John Abbott: *Peasants in the rural public. The Bavarian Bauernbund 1893–1933.* Chicago, University of Illinois, Dissertation, 2000 (englisch; insbes. über Versuche, den Antisemitismus im BB voranzubringen; nicht gedruckt).
- Hannsjörg Bergmann: *Der Bayerische Bauernbund und der Bayerische Christliche Bauernverein 1919–1928* (= *Schriftenreihe zur bayerischen Landesgeschichte.* Bd. 81). C. H. Beck, München 1986, ISBN 3-406-10482-7 (Zugleich: Regensburg, Universität, Dissertation, 1982/1983).
- Thomas Gräfe: *Bayerischer Bauernbund.* In: Wolfgang Benz (Hrsg.): *Handbuch des Antisemitismus. Judenfeindschaft in Geschichte und Gegenwart.* Band 5: *Organisationen, Institutionen, Bewegungen.* de Gruyter u. a., Berlin u. a. 2012, ISBN 978-3-598-24078-2, S. 54–56.
- Derek Hastings: *Catholicism and the Roots of Nazism. Religious Identity and National Socialism.* Oxford University Press, Oxford u. a. 2010, ISBN 978-0-19-539024-7.
- Heinz Haushofer: *Der Bayerische Bauernbund 1893–1933.* In: Heinz Gollwitzer (Hrsg.): *Europäische Bauernparteien im 20. Jahrhundert* (= *Quellen und Forschungen zur Agrargeschichte.* Bd. 29). Fischer, Stuttgart u. a. 1977, ISBN 3-437-50189-5, S. 562–586.
- Anton Hochberger: *Der Bayerische Bauernbund. 1893–1914* (= *Schriftenreihe zur bayerischen Landesgeschichte.* Bd. 99). C. H. Beck, München 1991, ISBN 3-406-10680-3.
- Alois Hundhammer: *Geschichte des Bayerischen Bauernbundes.* Pfeiffer, Regensburg 1924, (Zugleich: München, Universität, Dissertation, 1923).
- Johann Kirchinger: *Der Bauernrebell. Das Leben des streitbaren Landtagsabgeordneten Franz Wieland (1850–1901), Ökonom in Hierlbach, Post Straubing.* Pustet, Regensburg 2010, ISBN 978-3-7917-2271-9.

Weblinks [Bearbeiten | Quelltext bearbeiten]

- Oliver Braun: *Bayerischer Bauernbund (BB), 1895–1933*. In: *Historisches Lexikon Bayerns*
- Programm des Bayerischen Bauernbundes von 1895 (Dokument als PDF-Datei; 207 kB)
- Programm des Bayerischen Bauernbundes von 1920 (Dokument als PDF-Datei; 224 kB)
- *Bayerns Bauern während der Novemberrevolution 1918/1919: Ursachen und Grenzen der Revolutionierung einer bäuerlichen Bevölkerung*; Artikel von Ulrich Linse zur Rolle des Bayerischen Bauernbundes während der Novemberrevolution 1918/19 in Bayern

Einzelnachweise [Bearbeiten | Quelltext bearbeiten]

1. ↑ Stefan Primbs: *Schlachtsteuer – Historisches Lexikon Bayerns.* Abgerufen am 21. Juni 2019.

Normdaten (Körperschaft): GND: 2089793-5 | LCCN: n86033812 | VIAF: 263776465

Kategorien: Bayerische Geschichte (19. Jahrhundert) | Bayerische Geschichte (20. Jahrhundert) | Partei (Deutsches Kaiserreich) | Partei (Weimarer Republik) | Bauernpartei | Gegründet 1895 | Aufgelöst 1933

Figure 2: German Wikipedia Page on the Bavarian Peasant's League. Further readings highlighted.

4 A Wikipedia stub is an incomplete entry that lacks references. Do not rely on Wikipedia stubs because they are not reliable sources.

Following the link to the German Wikipedia entry reveals far more information with several references and further readings (Figure 2). I first tried to track down some of the books and dissertations, but could not access them online.

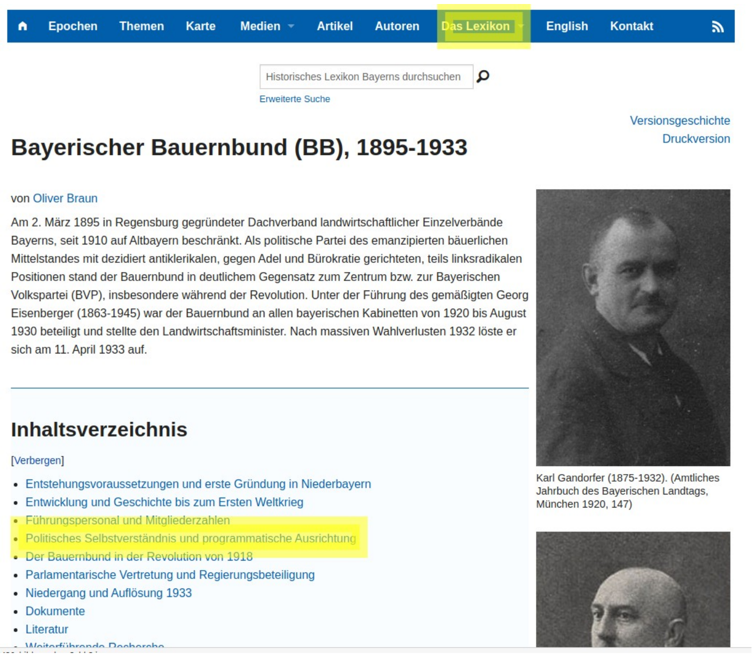

Figure 3: Historical Lexicon of Bavaria webpage for the Bavarian Peasants' League. Links to "About us" section and relevant information on party variables highlighted.

Next, I consulted the entries in the further readings section and found a detailed description of the party's history and its programmatic orientation in the Historical Lexicon of Bavaria (Figure 3). A quick look into the Lexicon's "About us" section (Impressum) revealed that it was administered by the Bavarian State Library, a trustworthy academic institution. In the "Political self-image and programmatic orientation" section (translated from German), the Lexicon provides the following relevant information:

> *Even if the Bauernbund movement initially came into being as an agrarian-medium-sized organization with primarily economic-political goals, it saw itself - in contrast to the Reichs-Landbund or the Christian farmers' associations - from the beginning as a political party with the aim of entering parliaments. The farmers' union appeared as a determined opponent and declared challenger to the established Center Party, the traditional political home of the Bavarian rural population. Like the center - from 1918 the Bavarian People's Party (BVP)- and the Christian farmers‘ associations, although firmly rooted in the rural-agrarian Catholic milieu, the farmers' union was decidedly anti-clerical and very critical of the nobility as well as the church and state authorities and the bureaucracy. It*

> *was therefore given the label "Social Democracy of the Flat Country" (Bergmann, Bauernbund, 19). The various farmers' federation programs from 1897 emphasized not only economic and agricultural policy demands, but also the independence of Bavaria, basic democratic principles, the separation of state and church, the nationalization of the school system and the abolition of religious school supervision. Against the background of these basic programmatic lines, however, the Bauernbund was shaped throughout its existence by internal party disputes between a moderate and a left, more radical wing.*[5]

Based on this source, I coded the relevant variables in Table 1 below.

If you do not speak the language of a Wikipedia article that seems to provide relevant information, use Google Translate and copy the Wikipedia URL into the left box. A link to the translated website will appear in the right box (see Figure 4). Alternatively, try DeepL.

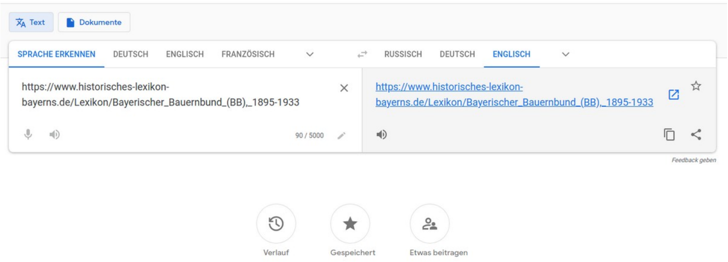

Figure 4: Google Translate webpage that translates the Historical Lexicon of Bavaria's entry for the Bavarian Peasants' League. Link to the translated version on the right.

5 Braun, Oliver (2006). „Bayerischer Bauernbund (BB), 1895-1933." *Historisches Lexikon Bayerns.* Accessed on 18 Mar 2021 at: http://www.historisches-lexikon-bayerns.de/Lexikon/Bayerischer_Bauernbund_(BB),_1895-1933

Table 1: Variable values for the Bavarian Peasants' League.

**Variable**	**Value**	**Evidence**
left_right	Center-left (2)	The descriptor "Social Democracy of the Flat Country" links the BPL to the Social Democratic Party of Germany – a center-left party. The statement, "the Bauernbund was shaped throughout its existence by internal party disputes between a moderate and a left, more radical left, more radical wing", highlights that the party is on the left of the political spectrum but its moderate wing seems to suggest not too radical an agenda.
rel_claim_flag	No (0)	The statement "the farmer's union was decidedly anti-clerical" and the goal to abolish "religious school supervision" provide evidence against a religious claim.
lin_claim_flag	Yes (1)	The party's programs emphasize the "the independence of Bavaria" – clear evidence of a claim to represent the linguistically distinct Bavarians.
eth_base_group	Bavarians	Both the name of the party (Bavarian Peasants' League) and its association with Bavarian farmers point to Bavarians as the key support group of the party.
rural_claim_flag	Yes (1)	The source describes the party as "firmly rooted in the rural-agrarian Catholic milieu" and as advancing "agricultural policy demands." These are clear indicators of a rural claim.
antisys	No (0)	The party supported basic democratic principles and does not fit the definition of an anti-system party.
violent_wing_name	NA	While the source does not explicitly describe the absence of violent wing, the description of the party allows one to conclude that it pursued its goals by non-violent, electoral means.
factions	moderate faction | radical-left faction	According to the source, the BPL experienced internal party disputes between a moderate and a left, more radical wing. We can thus report two factions.

References

Bakker, Ryan, Liesbet Hooghe, Seth Jolly, Gary Marks, Jonathan Polk, Jan Rovny, Marco Steenbergen & Milada Anna Vachudova. 2020. *1999 −2019 Chapel Hill Expert Survey Trend File*. Version 1.2.3 Available on chesdata.eu.

Boix, Carles, Michael K. Miller & Sebastian Rosato. 2013. "A Complete Data Set of Political Regimes, 1800-2007." *Comparative Political Studies* 46(12): 1523-54.

Boix, Carles, Michael K. Miller & Sebastian Rosato. 2018. *Boix-Miller-Rosato Dichotomous Coding of Democracy, 1800-2015*. Harvard Dataverse, V3. Available on https://doi.org/10.7910/DVN/FJLMKT.

Cagé, Julia & Thomas Piketty. 2023. *Une Histoire du Conflit Politique: Elections et Inégalités Sociales en France, 1789-2022*. Paris, Le Seuil.

Casal Bértoa, Fernando & Zsolt Enyedi. 2022. Who governs Europe? A new historical dataset on governments and party systems since 1848. *European Political Science* 21: 150–64.

Döring, Holger & Philip Manow. 2021. *Parliaments and governments database (ParlGov): Information on parties, elections and cabinets in modern democracies.* Development version. Available on parlgov.org.

Falter, Jürgen W. & Dirk Hänisch. 1990. *Wahl- und Sozialdaten der Kreise und Gemeinden des deutschen Reiches von 1920 bis 1933*. ZA8013 Datenfile Version 1.0.0.

Kollman, Ken, Allen Hicken, Daniele Caramani, David Backer & David Lublin. 2019. *Constituency-level elections archive*. Center for Political Studies, University of Michigan. Ann Arbor, MI.

Nohlen, Dieter & Philip Stöver. 2010. *Elections in Europe. A Data Handbook*. Baden-Baden: Nomos.

Popovic, Milos. 2020 *Yugoslavia in 1931: The census data on the county/town level*. v.1.0.0-November 2020

Singer, J. David & Melvin Small. 1994. *Correlates of War Project: International and Civil War Data, 1816-1992*. Inter-University Consortium for Political and Social Research: Ann Arbor, MI.

Walker, Brian M. 1992. *Parliamentary election results in Ireland: 1918-92: Irish elections to parliaments and parliamentary assemblies at Westminster, Belfast, Dublin, Strasbourg*. Dublin: Royal Irish Academy.
